# NAC transcription factors are key regulators of Brassinolide-Enhanced drought tolerance in Camellia oil tree

**DOI:** 10.1186/s12870-025-06653-0

**Published:** 2025-05-13

**Authors:** Kaizheng Lu, Yiyang Gu, YiXin Du, Yaxuan Yao, Xiaofeng Tan, Lichao Wu, Junqin Zhou, Jun Yuan

**Affiliations:** 1 Key Laboratory of Cultivation and Protection for Non-Wood Forest Trees, Ministry of Education, Central South University of Forestry and Technology, Changsha, 410004 China; 2 Academy of Camellia Oil Tree, Central South University of Forestry and Technology, Changsha, 410004 China; 3 College of Landscape Architecture, Central South University of Forestry and Technology, Changsha, 410004 China; 4 Tree Building, Central South University of Forestry and Technology, No. 498 Shaoshan South Road, Tianxin District, Changsha, Hunan 410004 China

**Keywords:** Camellia oil tree, Drought, Plant hormone, Brassinolide

## Abstract

Seasonal drought has hindered the sustainable growth of the Camellia oil tree industry. While brassinolide (BL) can mitigate drought stress in plants to some extent, the regulatory mechanisms underlying BL’s effects in Camellia oil tree remain unclear. To investigate the mechanisms by which BL alleviates drought stress in Camellia oil tree, three-year-old ‘Huashuo’ cutting seedling was exposed to three experimental treatments: CK (normal watering), UW (no watering), and BL (no watering, sprayed with 1 mg∙L^− 1^ BL). Leaf anatomical observation, hormone quantification, and transcriptomic profiling were performed on leaf samples collected at days 2, 4, and 6 following treatments. The results demonstrated that (**i**) BL treatment significantly increased leaf and spongy tissue thickness; (**ii**) BL reduced ABA and IAA levels in leaves under drought stress; (**iii**) WGCNA identified three modules linked to ABA, IAA, and their corresponding hormone levels, with subsequent analysis infer NAC transcription factors (TFs) as the primary regulators in these modules. This study contributes to a deeper understanding of the role of BL in alleviating drought stress in tree crop and provides a fundamental reference for molecular breeding and genetic improvement of Camellia oil tree.

**Supplementary Information:**

The online version contains supplementary material available at 10.1186/s12870-025-06653-0.

## Introduction

The Camellia oil tree is a species unique to China, primarily distributed in the hilly regions of southern China, and its seeds are an important source of high-quality edible oil [1, 2]. As of 2024, the planting area of Camellia oil trees reached approximately 4.87 million hectares, with a seed yield exceeding 3.3 million tons and an oil production of about 165,000 tons [3]. Taking advantage of the ability of Camellia oil tree to grow in hilly areas that are unsuitable for farmland can free up arable land currently used for oil crop cultivation, it provides an ecological solution to alleviate the competition between oil production and food production. This has important implications for ensuring global food security. However, the expansion of Camellia oil tree plantations faces a critical paradox: its crucial fruit development period (August-October) coincides with seasonal droughts exacerbated by subtropical monsoon climate variability, causing up over 15-34% yield losses in drought stress [1, 2, 4].

In the early stages of drought stress, plants reduce and streamline their leaves; the balance between palisade and spongy tissues diminishes, stomatal opening decreases, and a series of changes occur, all aimed at minimizing non-essential functions. If the plant is in the shoot elongation phase, drought inhibits its growth, resulting in shortened new shoots [5]. Prolonged drought stress leads to leaf and branch curling, desiccation, and eventual abscission, ultimately causing plant death. In addition to these phenotypic changes, plants respond to drought by initiating specific metabolic reactions, including alterations in enzyme activity and phytohormone levels [6, 7]. Drought stress increases the accumulation of reactive oxygen species (ROS) in plants. To mitigate the resulting damage, plants enhance the production of antioxidant enzymes such as superoxide dismutase (SOD), peroxidase (POD), ascorbate peroxidase (APX), and catalase (CAT), as well as non-enzymatic compatible solutes like proline and glutathione [8–10].

Brassinosteroids (BRs), first identified in oilseed rape (Brassica napus) in 1979, constitute the sixth class of plant hormones [11]. Brassinolide (BL), the most biologically active BR family member, was the first brassinosteroid to be isolated and characterized. BRs play crucial regulatory roles in diverse physiological processes including cell division, stomatal development, photosynthesis, and enzymatic activities [12]. Furthermore, BRs enhance plant tolerance to various abiotic stresses [13]. For example, BRs alleviate drought stress in tomato, poplar, and wheat by improving photosynthetic efficiency, enhancing antioxidant enzyme activities, and increasing endogenous ABA levels [14–16].

Three cell-surface receptors have been identified for BRs: BRASSINOSTEROID-INSENSITIVE 1 (BRI1), BRI1-LIKE 1 (BRL1), and BRL3 [17, 18]. Both BRI1 and BRL3 participate in drought stress responses, with BRI1 functioning as a negative regulator of BR-mediated drought tolerance [19, 20]. These receptors indirectly regulate downstream transcription factors through activation of BZR1/BES1 (BRI1 EMS SUPPRESSOR 1/BRASSINAZOLE RESISTANT 1), core components of BR signaling transduction, rather than directly modulating transcriptional regulators [21]. Notably, BR signaling pathways exhibit species-specific variations [22] and functional diversity across plant species [23]. Several transcription factors including WRKY46, WRKY54, WRKY70, bHLH (basic helix-loop-helix), and AP2/ERF (APETALA2/ETHYLENE RESPONSIVE FACTOR) have been implicated in BR-regulated drought responses [24–26]. The NAC (NO APICAL MERISTEM – ARABIDOPSIS TRANSCRIPTION ACTIVATION FACTOR – CUP-SHAPED COTYLEDON) TF family, one of the largest plant-specific TF families, shows strong associations with drought stress [27]. However, no evidence currently links NAC TFs to BR signaling pathways in drought responses. The application of BRs in Camellia oil trees remains understudied. Current research demonstrates that foliar BR application enhances nitrogen (N), phosphorus (P), and potassium (K) content in spring shoot leaves while increasing oil yield [28]. Nevertheless, their potential role in mitigating drought stress in this economically important species remains unexplored.

Given the unresolved role of NAC TFs in BR-mediated drought regulation and the uncertainty surrounding BR-mediated drought responses in Camellia oil tree, this study was conducted using Camellia oil tree saplings subjected to varying drought conditions. Through analyses of leaf anatomical structures, endogenous hormone levels, and time-series gene expression, we investigated whether BRs alleviate drought stress in Camellia oil tree and determined whether NAC TFs participate in BR signaling pathways. The research ultimately seeks to establish mechanistic links between BR signaling and NAC-mediated drought responses while providing theoretical foundations for the sustainable expansion of Camellia oil tree cultivation and the development of drought-resistant varieties.

## Materials and methods

### Plant material

The experimental material consisted of three years old cutting saplings of ‘Huashuo’, a nationally recognized high-yielding variety of the Camellia oil tree [29]. The substrate used was red clay, sourced from Camellia oil tree plantations and sieved prior to use. The experiment was conducted at the nursery of Central South University of Forestry and Technology (Changsha, Hunan Province, China). Before the experiment began, all Camellia oil tree saplings underwent a 10-month pre-cultivation period, during which uniform water and fertilizer management practices were implemented. Upon completion of this period, saplings exhibiting consistent growth were selected for the experiment. The experiment began on August 20, 2022, and included three treatments: (**i**) CK, normal water management; (**ii**) UW, no water applied; and (**iii**) BL, no water applied with the addition of 1 mg∙L⁻¹ of BL (Macklin, Shanghai). The BL concentration was selected based on previous studies by Su et al. [30] and Zheng et al. [31], in which three concentrations (0.5 mg∙L⁻¹, 1.0 mg∙L⁻¹, and 1.5 mg∙L^− 1^) were tested, with 1.0 mg∙L^− 1^ determined to be the most appropriate (unpublished data). Each treatment was replicated three times, with each replicate consisting of five Camellia oil tree saplings. The temperatures recorded during the experiment are provided in Table S1.

Leaves from the second to third positions from the tips of the branches were collected on day 2 (D2), day 4 (D4), and day 6 (D6) after the initiation of the experiment. The samples were immediately wrapped in tin foil, snap-frozen in liquid nitrogen, and subsequently stored at − 80 °C for further analysis of the index. Soil moisture levels during the experiment are detailed in Table S2.

### Determination of leaf and soil water content

Soil water content was measured using a soil thermohygrometer (S21AN, Pengyun, China), while leaf water content was determined using the drying and weighing method. The collected leaves were individually weighed using an analytical balance with an accuracy of 0.001 g to record their initial fresh weight. The leaves were then fully submerged in deionized water for 24 h. After removal, residual surface moisture was blotted dry using absorbent paper, and the leaves were subsequently weighed to record their saturated weight. The leaves were then placed in envelopes and dried to a constant weight. After drying, the leaves were weighed again, water content (WC) and relative water content (RWC) were calculated using the following formulas:

$$\:WC=(Fw-Dw)/Fw\times\:100\%\:\:\left(1\right)$$

where *WC* is the water content (%), *Fw* is the fresh weight (g), and *Dw* is the dry weight (g).

$$\:RWC=(Sw-Fw)/Sw\times\:100\%\:\:\left(2\right)$$

where *RWC* is the relative water content (%), *SW* is the weight of after absorbing water for 24 h (g), *Fw* is the fresh weight (g).

### Photosynthesis measurements

Measurements were conducted in the experimental plots using the portable photosynthesis measurement system LI-6400 (LI-COR, Lincoln, Nebraska, USA) between 08:00 and 11:00 on day 2, day 4, and day 6 of the experiment. During the measurements, the leaf chamber environment was left uncontrolled, with no regulation of CO₂ concentration, photosynthetically active radiation (PAR), or temperature, and natural light was used as the light source. Gas exchange was facilitated by connecting the buffer bottle to the air inlet of the main system. In each experimental plot, five plants with vigorous growth and no signs of diseases or pests were selected. Photosynthetic parameters were measured on three leaves from each plant. Experiments were conducted on days 2, 4, and 6 after the start of the study.

### Paraffin anatomical

Freshly harvested leaves were dissected into rectangles along the leaf veins using a razor blade and then fixed in FAA solution (composed of 5% (v/v) glacial acetic acid, 5% (v/v) formalin, and 70% (v/v) ethanol). The samples were subjected to vacuum infiltration for 1 h. Following fixation, the leaves were dehydrated sequentially with ethanol (50%: 3–5 s, 70%: 3–5 s, 80%:3–5 s) and xylene. Subsequently, the samples were infiltrated with paraffin wax at 60 °C and embedded into paraffin blocks. Sections were prepared using a slicer (LEICA RM2016, Germany) and stained with Senna solid green following dewaxing in xylene. Finally, images of the sections were captured by scanning under a microscope (Nikon Eclipse E100, Japan) equipped with an imaging system (NIKON DS-U3, Japan).

Measurements of palisade tissue and spongy tissue were conducted using Fiji [32] in windows 10 desktop computer. In summary, the vertical length of each organ was measured using the Line tool in Fiji, following scale calibration. Ten measurements were taken per section to ensure relatively accurate results.

### Determination of hormone

The 100 mg of powdered leaves were accurately weighed and combined with 1000 µL of extraction solution (methanol: acetonitrile: water = 2:2:1, v/v). The mixture was subjected to ultrasonication for 10 min in an ice-water bath with shaking. Subsequently, the sample was snap-frozen in liquid nitrogen for 1 min, a process repeated three times, followed by incubation at -20℃ for 1 h. Afterward, the mixture was centrifuged at 13,000 rpm for 15 min at 4℃, and the supernatant was carefully collected. The supernatant was then evaporated using a nitrogen blower until dryness. To reconstitute the residue, 100 µL of acetonitrile: water (1:1, v/v) was added, followed by 30 s of shaking. The solution underwent further ultrasonication for 10 min in an ice-water bath and was centrifuged again at 13,000 rpm for 15 min. The resulting supernatant was subjected to analysis, with the content determined based on the peak area obtained.

Chromatographic analysis was performed using an ACQUITY UPLC^®^ BEH C18 column (2.1 × 100 mm, 1.7 μm, Waters, USA). The injection volume was 5 µL, and the column temperature was maintained at 40℃. The mobile phase consisted of solvent A (25 mM CH_3_COONH_4_ + 25 mM NH_4_OH in 100% H_2_O) and solvent B (100% ACN). Gradient elution conditions were as follows: 0–1 min, 85% B; 1–12 min, linear decrease from 85 to 65% B; 12–12.1 min, linear decrease from 65 to 40% B; 12.1–15 min, hold at 40% B; 15–15.1 min, linear increase from 40 to 85% B; 15.1–20 min, hold at 85% B. The flow rate was maintained at 0.3 mL/min.

Mass spectrometric analysis was performed using an electrospray ionization (ESI) source. The ion source temperature was set at 600℃, and the ion source voltage was either − 4500–5500 V. The air curtain gas was set to 20 psi, while both the atomization gas and auxiliary gas were set to 60 psi. Scanning was conducted using multiple reaction monitoring (MRM) mode.

### Construction of RNA-seq libraries

Total RNA extraction was carried out using a Plant RNA Kit from Omega (USA). The concentration and purity of the RNA samples were assessed using a Nanodrop 2000 spectrophotometer from Thermo Fisher Scientific and an Agilent 2100 Bioanalyzer equipped with a 2100 RNA Nano 6000 assay kit from Agilent Technologies.

For transcriptome sequencing library preparation, poly-A enriched RNA from eukaryotic total RNA was obtained using the TIANSeq mRNA Capture Kit from TIANGEN. Subsequently, the TIANSeq Fast RNA Library Kit from Illumina was employed to construct the transcriptome sequencing libraries. This process involved RNA fragmentation, cDNA synthesis of both strand 1 and strand 2, end repair, A-tailing, ligation of sequencing adapters, size selection, and library PCR enrichment. Following library preparation, the concentration of the libraries was quantified using a Qubit 2.0 fluorometer from Life Technologies. The libraries were then diluted to a concentration of 1 ng/µl before determining the insert size on an Agilent 2100 Bioanalyzer. Library quantification with greater accuracy was performed using quantitative PCR (q-PCR) to ensure that the library activity exceeded 2 nM.

Clustering of the index-coded samples was conducted on a cBot Cluster Generation System using the TruSeq PE Cluster Kit v3-cBot-HS from Illumina as per the manufacturer’s instructions. Subsequently, the library preparations were sequenced on an Illumina sequencing platform, generating 150 bp paired-end reads.

An index of the reference genome was constructed, and the paired-end clean reads were aligned to the reference genome (Camellia oil tree ‘Huashuo’, unpublished data) using HISAT 2.2.4 [33].

### Gene expression analysis

Differential expression analysis of the two groups was conducted using the DESeq2 R package (version 1.16.1) [34]. The resulting P-values were adjusted via the Benjamini-Hochberg procedure to control the false discovery rate. Genes meeting the criteria of an adjusted *P* < 0.05 and|fold change| ≥ 2, as identified by DESeq2, were designated as differentially expressed genes (DEGs).

### Analyses of gene ontology and kyoto encyclopedia of genes and genomes

Gene Ontology (GO) and Kyoto Encyclopedia of Genes and Genomes (KEGG) analyses were conducted using TBtools [35]. Subsequently, the results were visualized by generating plots with ggplot2 in R (version 4.3.2).

### Weighted correlation network analysis (WGCNA)

Gene co-expression network analysis was performed in R using the WGCNA package [36]. The soft threshold was set to 0.8, and the Power value was set to 9. A hierarchical clustering tree was constructed for the DEGs within the network using the difference matrix between genes. The DEGs were then assigned to different modules using dynamic clipping, with default parameters set to a minimum module size of 10 and a cuttree height of 0.25. The resulting information on the modules of interest was exported for visualization in Cytoscape 3.10.1 [37]. Furthermore, chord diagrams depicting the relationships within the modules were generated in R by calling the circlize package [38].

### Quantitative real-time qRT-PCR validation

Selecting nine genes to confirm RNA-seq date by qRT-PCR. The primers were designed using Primer-BLAST (https://www.ncbi.nlm.nih.gov/tools/primer-blast/). After primer were designed, the specificity of the primers was checked in NCBI-BLAST (https://blast.ncbi.nlm.nih.gov/Blast.cgi), and primers with higher specificity were selected for use. The primers are listed in Table S3. Synthesis of cDNA from total RNA was performed using a reverse transcription kit (Vazyme, China). The final product of the reverse transcription reaction was diluted and used for qRT-PCR analysis. The qRT-PCR was performed with three biological replicates, and each sample was analyzed at least in triplicate and normalized using *CoGAPDH* (forward primer 5’- CTACTGGAGTTTTCACCGA-3’ and reverse primer 5’- TAAGACCCTCAACAATGCC-3’) as the reference genes [39]. The qRT-PCR amplification data were analyzed using the 2^−∆∆CT^ method [40].

### Data analysis

The data were compiled using Microsoft 365 (Microsoft, USA). Analysis of variance (ANOVA) was conducted for significance testing in SPSS 24.0 (IBM, USA) utilizing the Anova function. Post-hoc comparisons were performed using the Duncan test, with significant differences considered at the *P* < 0.05 level. All bar graphs were created using OriginPro 2023b (OriginLab Corporation, USA) for visualization.

## Results and analysis

### BL reduced the rate of decline in leaf water content under drought conditions

The leaf water content (LWC) under different treatments is presented in Table 1. At D2 and D4, no significant difference was observed in LWC between the UW and BL groups, but both were significantly lower than that of CK. By D6, the LWC of UW became significantly lower than those of CK and BL, while no significant difference persisted between CK and BL.

The relative leaf water content (RWC) of UW exhibited a progressive decline from D2 to D6. At D6, the RWC of UW was 29% and 24% lower than those of CK and BL, respectively.

**Table 1:** Leaf water content under different treatments

Index	Date	D2	D4	D6
Leaf water content m/m %	CK	59.21 ± 0.92a	62.35 ± 1.70a	59.32 ± 2.10a
	UW	57.75 ± 2.51a	58.59 ± 6.01a	50.07 ± 3.05b
	BL	58.41 ± 1.40a	57.77 ± 1.94a	57.33 ± 0.71a
Leaf relative water content %	CK	74.64 ± 2.20a	76.23 ± 1.29a	71.52 ± 0.45a
	UW	71.29 ± 6.05a	65.96 ± 3.15b	50.67 ± 2.63c
	BL	67.33 ± 3.93a	64.70 ± 0.27b	66.27 ± 1.83b

Note: Mean ± SD, different lowercase letters indicate significant differences among treatments at *P* < 0.05

### BL mitigates drought-induced decrease in photosynthetic rate

**Fig. 1:**
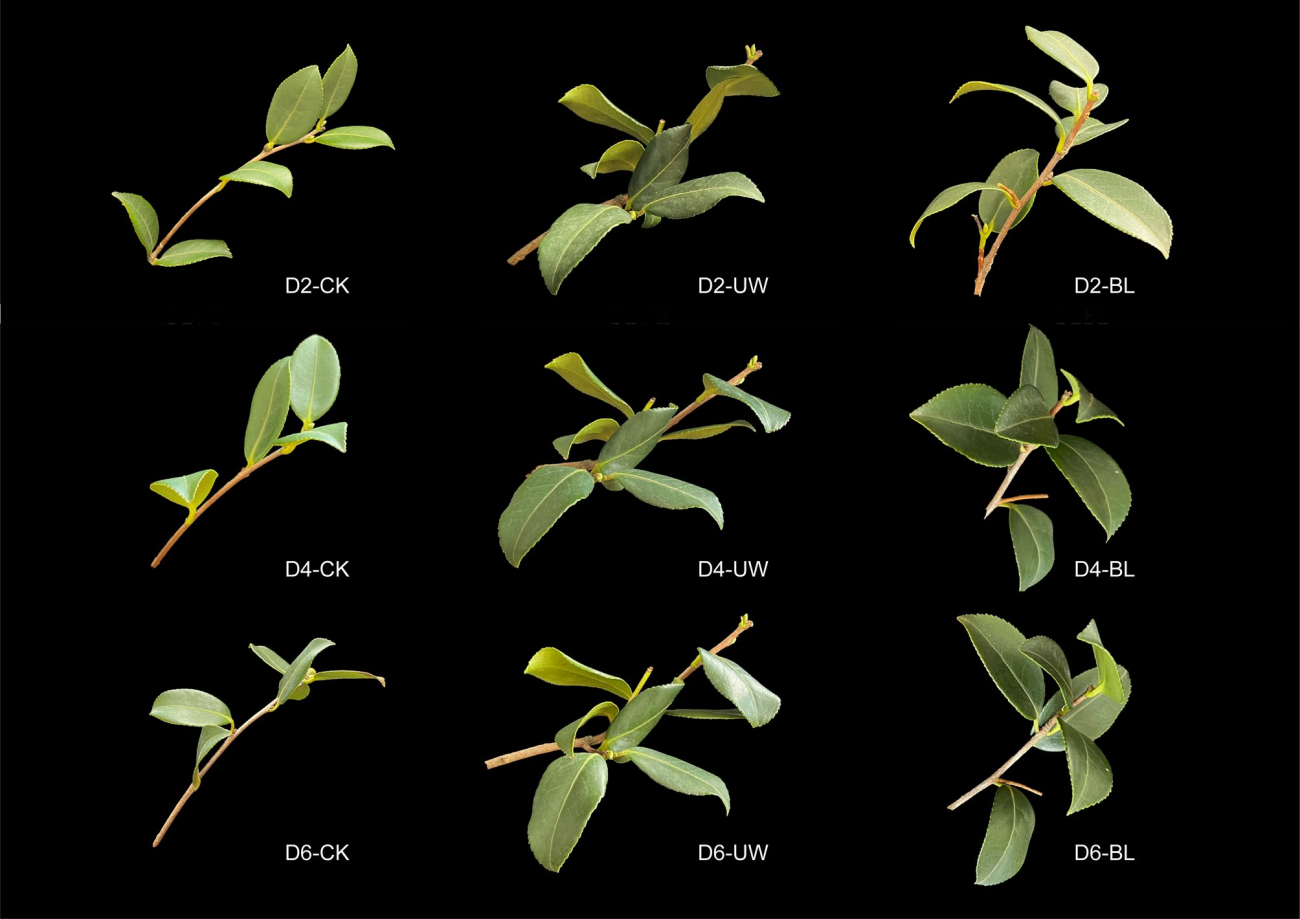
Camellia oil tree leaves with different treatments in different period

Leaves of the UW treatment began curling by D2 after treatment, with the severity of curling progressively intensifying over time (Fig. 1). Photosynthesis performance across treatments on D2, D4, and D6 revealed a significant suppression of the photosynthetic rate (Pn) in UW-treated leaves (Table 2). At D2 and D4, CK exhibited a photosynthetic rate 1.9 times higher than UW, with values of 8.64 µmol·m⁻²·s⁻¹ and 3.44 µmol·m⁻²·s⁻¹, respectively. In contrast, no significant difference in Pn was observed between BL and CK during these periods. By D6, CK maintained a photosynthetic rate 1.7 times greater than UW, while BL showed a notable decline compared to CK. Intercellular CO₂ concentrations (C_i_) in UW-treated leaves at D2, D4, and D6 were 329.9 µmol·mol⁻¹, 315.81 µmol·mol⁻¹, and 241.26 µmol·mol⁻¹, respectively, consistently the highest across all sampling periods; however, no significant differences were detected among treatments. Stomatal conductance (G_s_) and transpiration rate (T_r_) displayed no consistent trends across treatments or time points.

**Table 2:** Differences in photosynthesis of Camellia oil tree with different treatments

		Pn µmol∙m^− 2^∙s^− 1^	Gs mol∙m^− 2^∙s^− 1^	Ci µmol∙mol^− 1^	Tr mmol∙m^− 2^∙s^− 1^
D2	CK	8.64 ± 0.34a	0.17 ± 0.01a	319.63 ± 0.32a	3.68 ± 0.16a
	UW	4.54 ± 0.96b	0.10 ± 0.03b	329.90 ± 12.93a	2.52 ± 0.63b
	BL	7.67 ± 0.54a	0.16 ± 0.04ab	320.24 ± 15.27a	3.39 ± 0.67ab
D4	CK	3.44 ± 0.16a	0.04 ± 0.02a	224.43 ± 82.87a	1.36 ± 0.56a
	UW	1.78 ± 0.28b	0.04 ± 0.02a	315.81 ± 37.79a	1.31 ± 0.61a
	BL	3.07 ± 0.06a	0.05 ± 0.02a	286.80 ± 37.70a	1.47 ± 0.52a
D6	CK	8.30 ± 0.25a	0.07 ± 0.02a	188.77 ± 64.56a	2.28 ± 0.62a
	UW	4.84 ± 0.34c	0.05 ± 0.01ab	241.26 ± 26.43a	1.94 ± 0.41a
	BL	7.26 ± 0.16b	0.04 ± 0.01b	92.29 ± 40.90b	1.46 ± 0.21a

Note: Mean ± SD, different lowercase letters indicate significant differences at *P* < 0.05

### BL can increase the proportion of palisade and spongy tissue in leaves under drought conditions

**Fig. 2:**
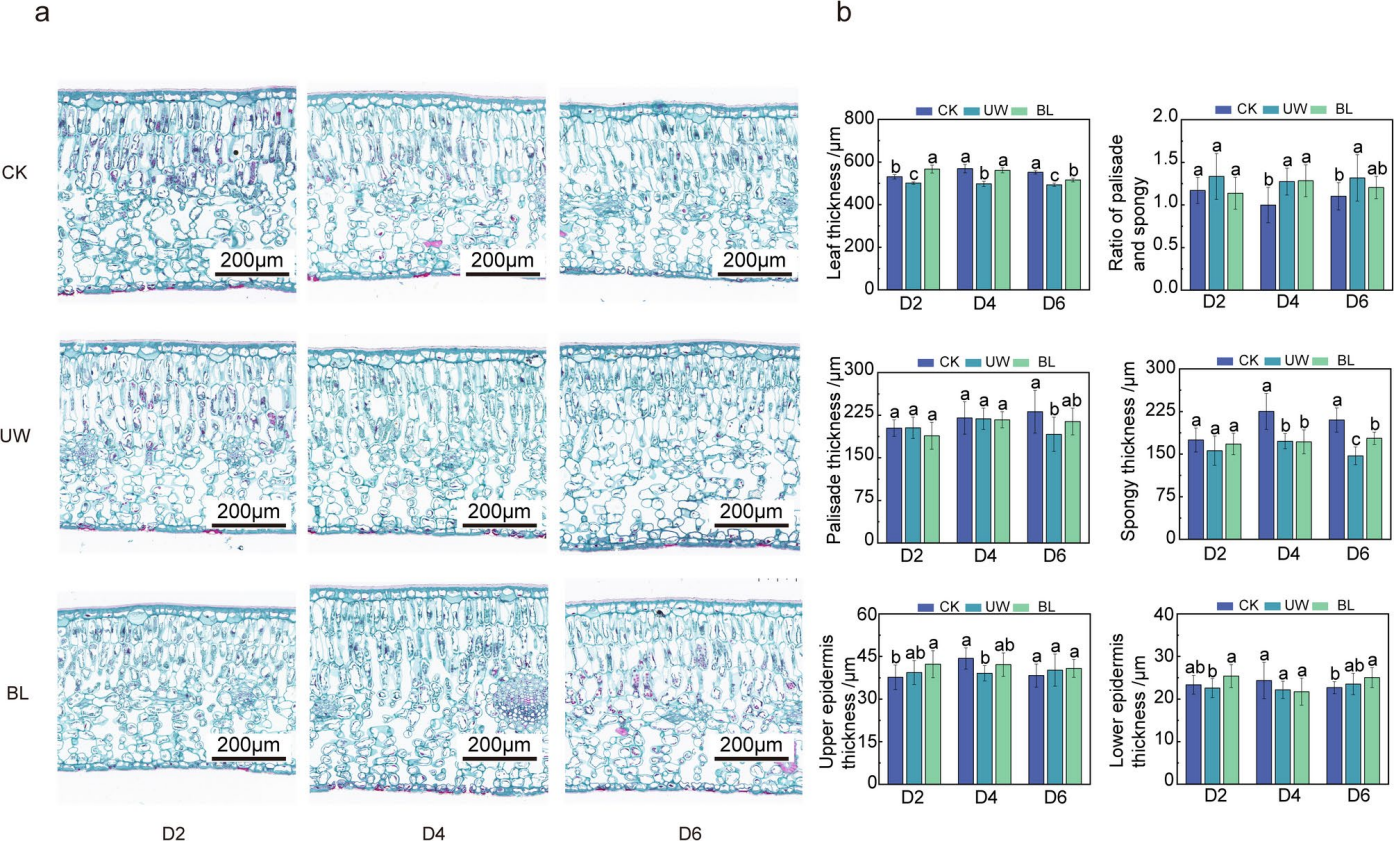
Differences in leaf structure of Camellia oil tree with different treatments. Note: (**a**) Paraffin sections from day 2, day 4, and day 6, respectively; (**b**) The results of measurements and statistics of the sections using Fiji, different lowercase letters indicate significant differences at *P* < 0.05

Drought stress led to a significant reduction in leaf thickness, with the same time, the ratio of palisade and spongy tissues in the UW was increased (Fig. 2). Examination of leaf slices at different time points revealed notable differences in leaf blade thickness in D2, with the UW exhibiting the thinnest leaf blades at 501.47 μm. By D4, leaf thickness in the CK and BL was not significantly different, but both were significantly greater than that of the UW. Moreover, the spongy tissue thickness in the CK was significantly greater than that of the UW and BL, measuring 225.12 μm. Additionally, the ratio of palisade tissue to spongy tissue in the CK was significantly lower compared to the UW and BL, with no significant difference observed between the latter two. By D6, significant differences in leaf thickness were observed among the CK, UW, and BL, with the CK displaying the greatest thickness followed by BL and UW. Similar trends were observed for spongy and palisade tissues. Notably, the ratio of palisade to spongy tissues in the UW treatment was the highest at 1.32, which was not significantly different from the BL but significantly higher than the CK. The upper and lower epidermis did not show clear trends across treatments.

### Significant differences in the hormone content of leaves under different treatments

**Fig. 3:**
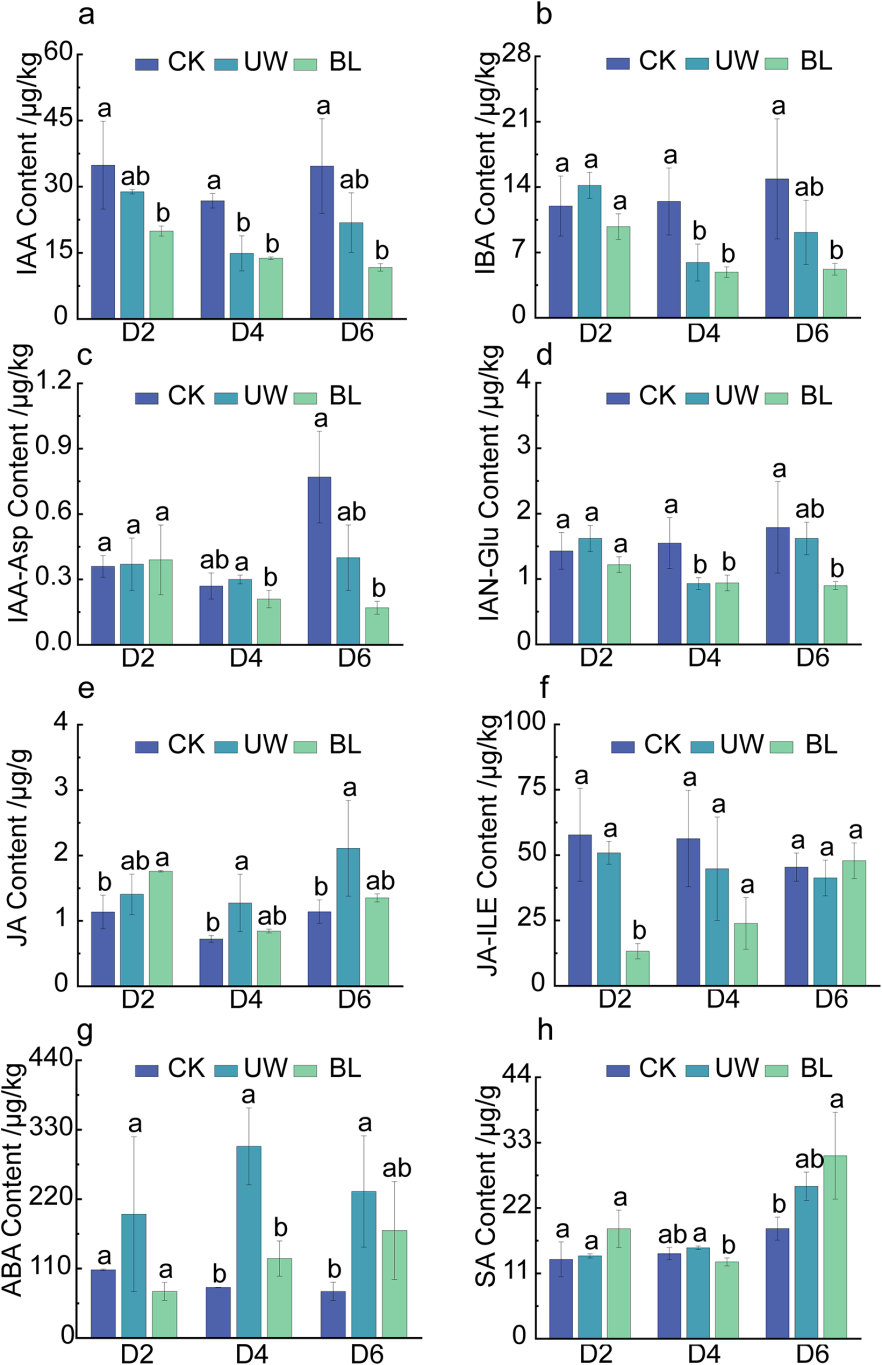
Hormone contents in leaves with different treatments. Note: Hormone levels in (**a**), (**b**), (**c**), (**d**), (**f**) and (**g**) are in µg/kg and (**e**), (**h**) are in µg/g, different lowercase letters indicate significant differences at *P* < 0.05

Hormone content analysis of the leaves revealed that abscisic acid (ABA) exhibited the most pronounced changes among the measured hormones (Fig. 3). At D4 and D6, ABA levels in UW were significantly elevated compared to CK, reaching 306.62 µg/kg and 232.12 µg/kg, respectively, representing 3.8- and 3.2-fold increases relative to CK. In contrast, no significant difference in ABA content was detected between CK and BL. Jasmonic acid (JA) content in UW also significantly surpassed that of CK, with values of 1.27 µg/g and 2.11 µg/g at D4 and D6, respectively, while BL and CK showed no significant divergence. Jasmonoyl-isoleucine (JA-ILE) content remained consistent across treatments, except for a transient reduction observed exclusively in BL at D2. The IAA-like hormones (IAA, IBA, IAA-ASP, and IAN-Glu) collectively displayed a unified trend: BL treatment markedly reduced auxin hormone concentrations in camellia oil tree leaves, with concentrations across treatments following the order CK > UW > BL.

### Transcriptome analysis identified drought-stress-regulated DEGs

Leaf samples from the three treatments were harvested at D2, D4, and D6 for transcriptome sequencing (RNA-seq) using the Illumina platform. Due to the lack of significant structural or hormonal divergence among treatments at D2, only data from D4 and D6 were retained for transcriptomic analysis. A total of 18 transcriptome libraries were generated, with three biological replicates per treatment at each time point. To identify DEGs, two comparative groups (CK vs. UW and UW vs. BL) were established at D4 and D6, respectively, followed by downstream functional analysis.

**Fig. 4:**
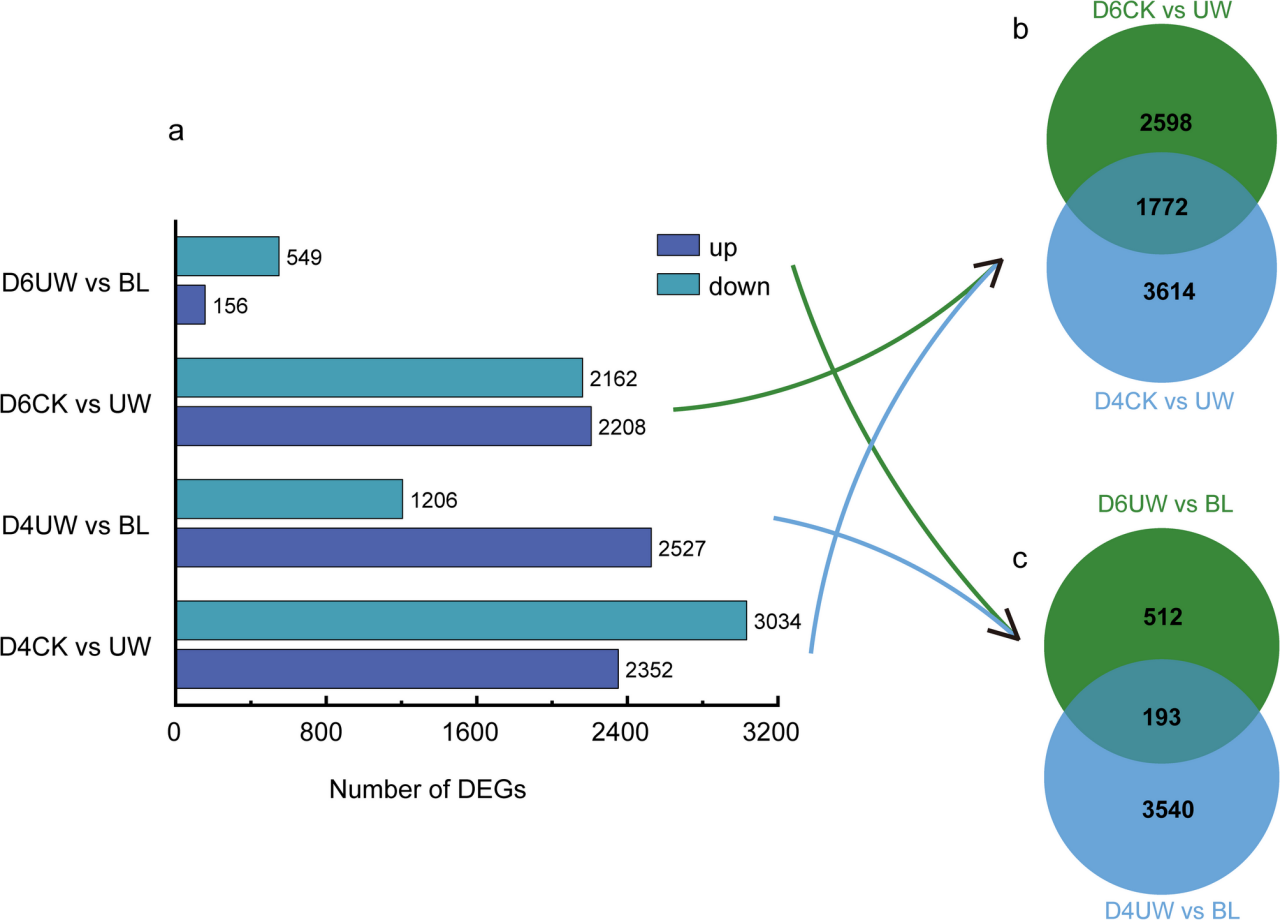
DEGs with different treatments

After filtering, a total of 5,386 DEGs (2,352 up-regulated and 3,035 down-regulated), 4,370 DEGs (2,527 up-regulated and 1,206 down-regulated), 3,733 DEGs (2,208 up-regulated and 2,162 down-regulated), and 705 DEGs (158 up-regulated and 549 down-regulated) were identified across the four comparison groups, respectively (Fig. 4a).

The D4 CK vs. UW and D6 CK vs. UW groups shared 1,772 genes, with 3,614 genes unique to D4 CK vs. UW and 2,598 genes unique to D6 CK vs. UW. Similarly, the D4 UW vs. BL and D6 UW vs. BL groups shared 193 genes, while 3,540 genes were exclusive to D4 UW vs. BL and 512 genes to D6 UW vs. BL.

To minimize confounding effects from drought duration-dependent gene expression, treatment-exclusive genes from the same comparison groups across D4 and D6 were pooled and deduplicated. These composite gene sets were then used for subsequent GO and KEGG enrichment analyses. As shown in Fig. 4b, the CK vs. UW group included 7,984 genes (2,598 + 1,772 + 3,614), while the UW vs. BL group contained 4,245 genes (512 + 193 + 3,540) (Fig. 4c).

### GO and KEGG enrichment analysis of drought-related DEGs

**Fig. 5:**
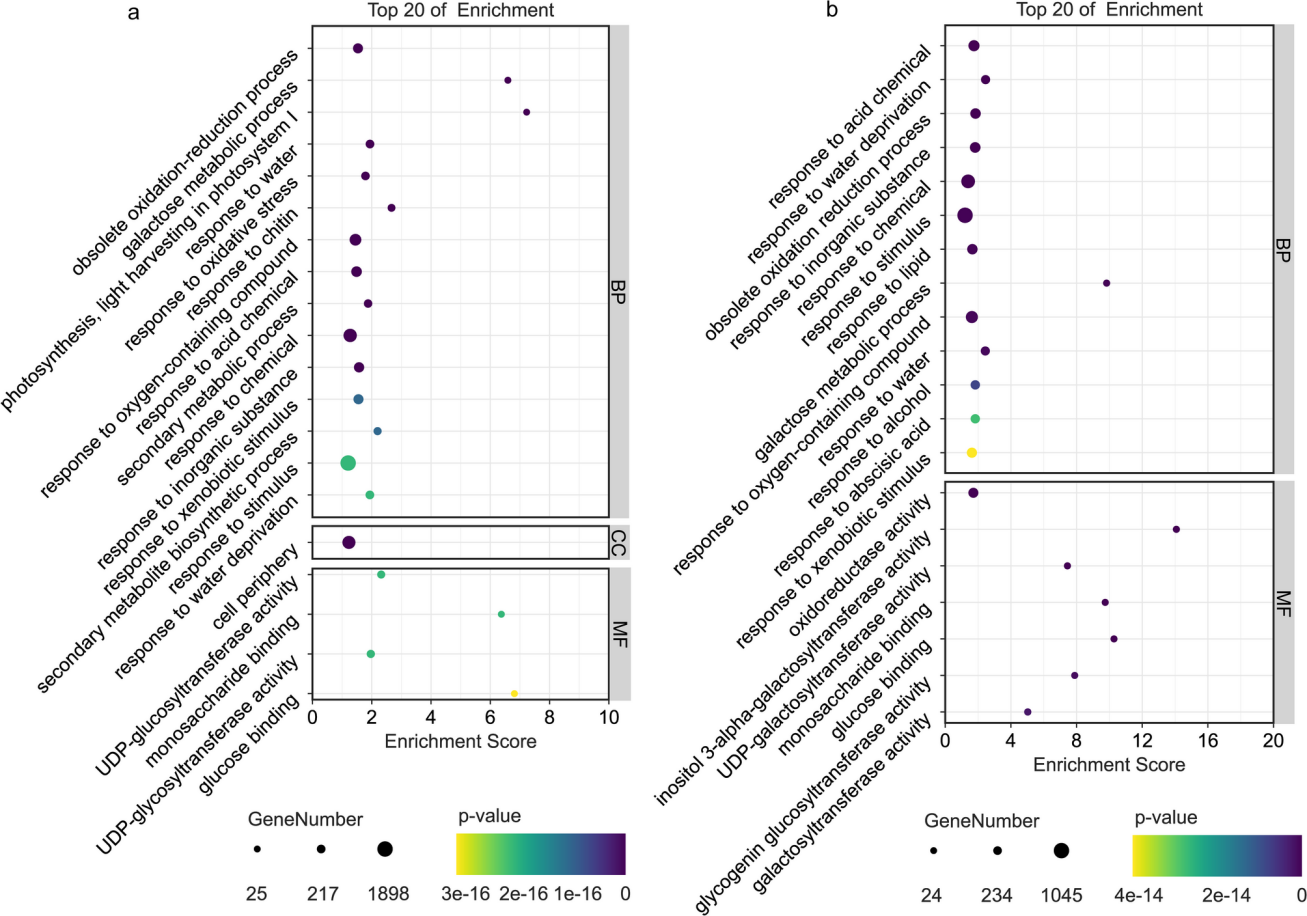
CK vs. UW (**a**) and UW vs. BL (**b**) differential gene Go enrichment analysis (Top 20). Note: BP stands for Biological Process, CC for Cellular Component, and MF for Molecular Function. The hue of each dot on the pathway chart corresponds to the magnitude of its p-value, where a darker shade signifies a smaller p-value. The size of the dot represents the quantity of differentially expressed genes enriched within that pathway, with larger dots denoting a higher number of enriched genes. The charts for BP, CC, and MF are each sorted individually

The two composite gene sets (CK vs. UW and UW vs. BL) underwent Gene Ontology (GO) functional annotation, with the top 20 enriched categories (ranked by p-value) visualized in bubble plots. In the CK vs. UW group, differentially expressed genes (DEGs) were predominantly enriched in metabolic processes (e.g., secondary metabolism, galactose metabolism) and chemical response pathways (e.g., response to acidic compounds, chitosan, and water deficit) (Fig. 5a). Conversely, DEGs in the UW vs. BL group were primarily associated with enzymatic activities (e.g., oxidoreductase activity, glycogenin glucosyltransferase activity) and responses to chemical stimuli (e.g., inorganic substances, lipids, abscisic acid) (Fig. 5b).

**Fig. 6:**
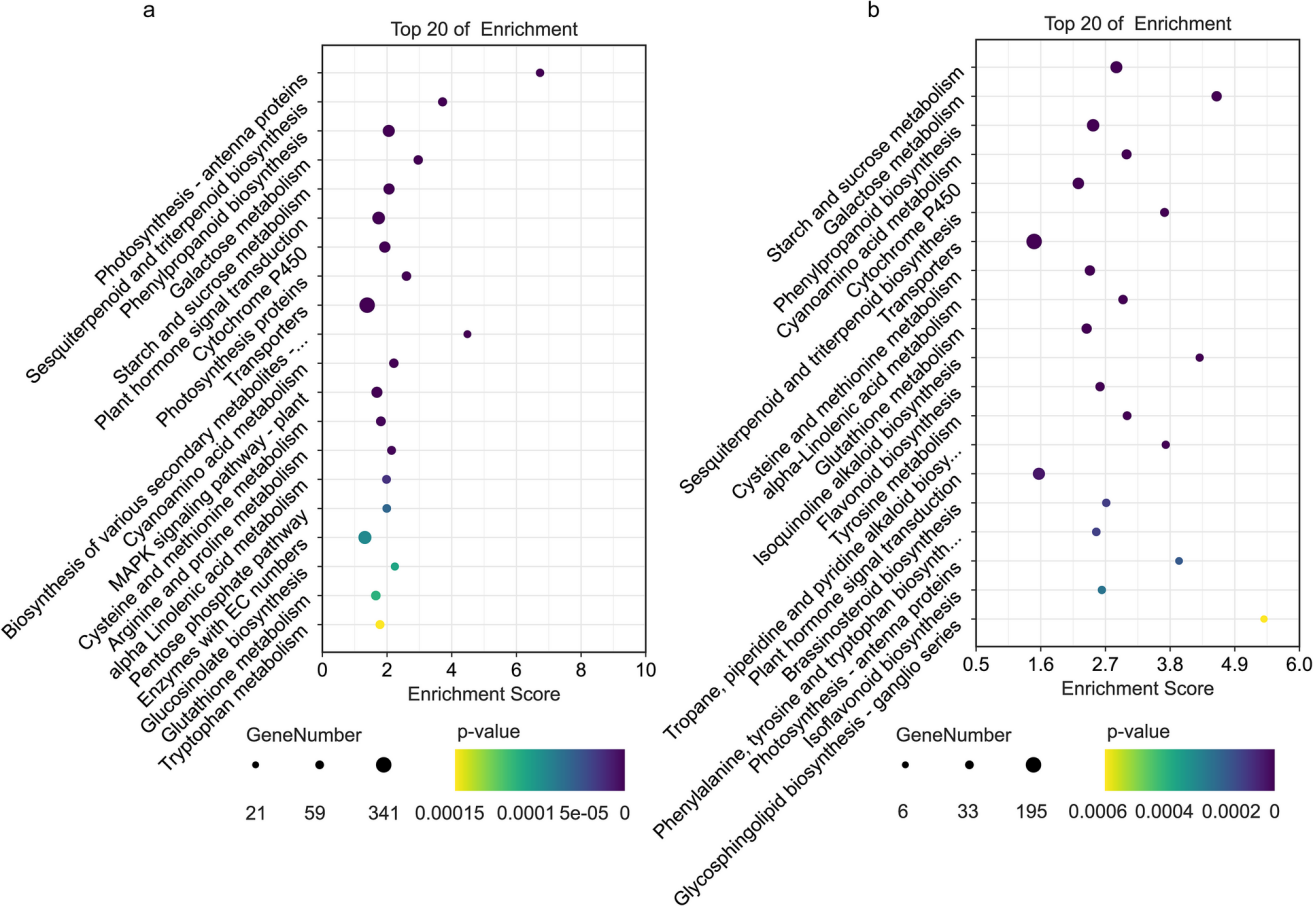
CK vs. UW (**a**) and UW vs. BL (**b**) differential gene KEGG enrichment analysis (Top 20). Note: The hue of each dot on the pathway chart corresponds to the magnitude of its p-value, where a darker shade signifies a smaller p-value. The size of the dot represents the quantity of differentially expressed genes enriched within that pathway, with larger dots denoting a higher number of enriched genes. The charts for a and b are arranged in ascending order of p-value size

To identify pathways linked to drought stress adaptation and BL-mediated mitigation, all DEGs were subjected to KEGG enrichment analysis. In the CK vs. UW group, the three most significantly enriched pathways (ranked by p-value and enrichment score) were photosynthesis-antenna proteins (32 genes), biosynthesis of various secondary metabolites (21 genes), and sesquiterpenoid and triterpenoid biosynthesis (49 genes) (Fig. 6a). For the UW vs. BL group, the top five enriched pathways included galactose metabolism (47 genes), glycosphingolipid biosynthesis—ganglio series (6 genes), isoquinoline alkaloid biosynthesis (16 genes), photosynthesis-antenna proteins (10 genes), and tropane, piperidine, and pyridine alkaloid biosynthesis (17 genes) (Fig. 6b).

### DEGs involved in plant hormone signal transduction

**Fig. 7:**
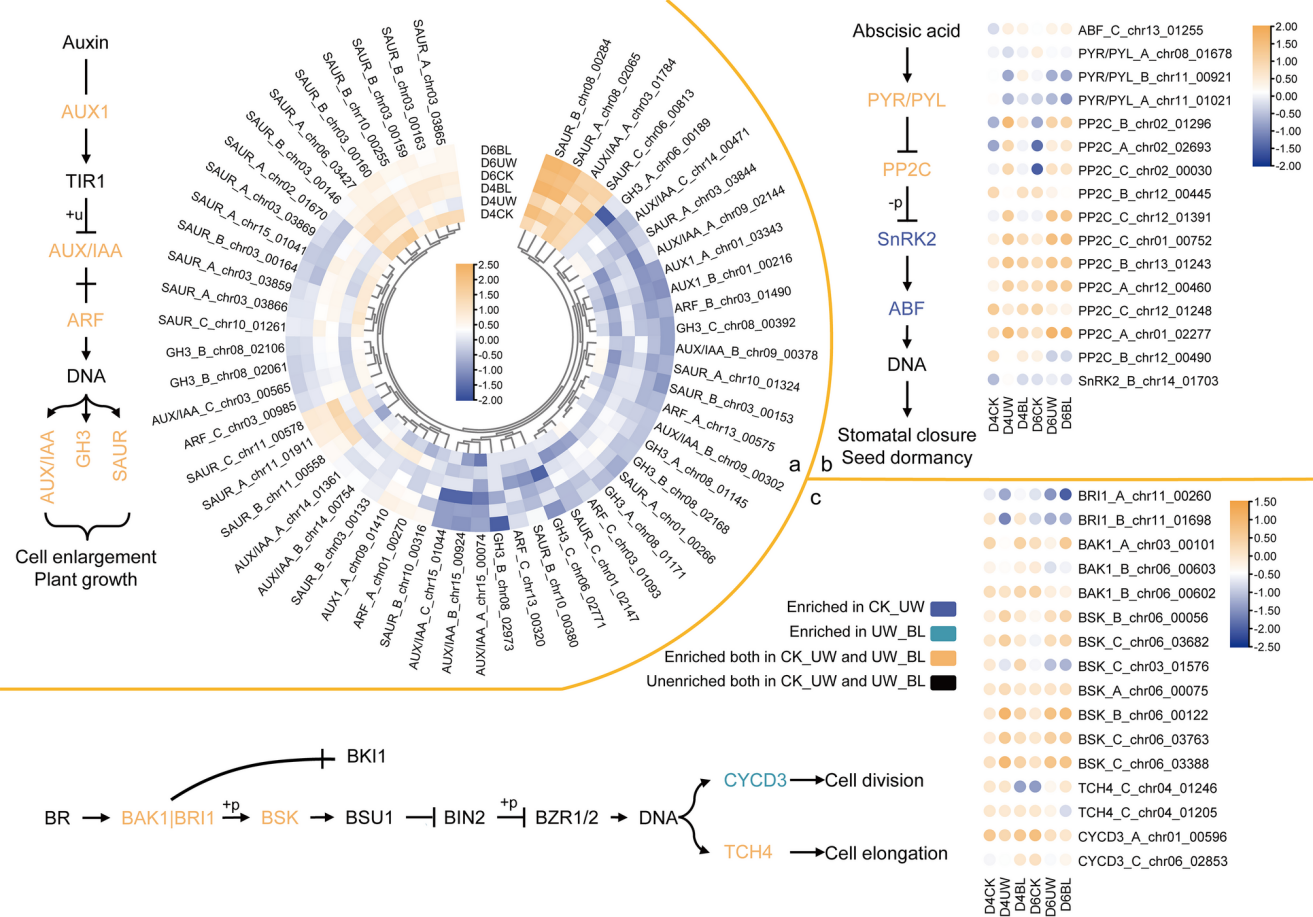
Gene enrichment in Auxin (**a**), ABA (**b**) and BR (**c**) transduction. Note: heatmap of DEGs associated with Auxin (**a**), ABA (**b**) and BR (**c**). Genes expression was based on mean FPKM(fragments per kilobase of transcript per million mapped reads) value from three biological replicates, which were log_2_ transformed and normalized. Signaling pathways of Auxin (**a**), ABA (**b**) and BR (**c**), different colors of the pathway indicate different sources of genes enriched in the linkage; The values represented by the colors are FPKM values after normalization (lg); “+p” and “-p” refer to phosphorylation events

To investigate the role of the phytohormone signaling pathway (K04075) in the response of the camellia oil tree to drought and the mitigation of drought stress by BL, the signaling pathways of Auxin, ABA, and BR were mapped (Fig. 7). On D4 post-treatment, drought resulted in down-regulation of *AUX1*,* ARF*,* GH3*, and *SAUR* in the Auxin pathway. In the ABA pathway, UW down-regulated *PYR/PYL_A_chr08_01678*,* PYR/PYL_B_chr11_00921*,* PYL_A_chr11_01021*,* PP2C_B_chr12_00445*,* PP2C_C_chr12_01248*, and *PP2C_B_chr12_00490*, while up-regulating *PP2C_B_chr02_01296*,* PP2C_A_chr02_02693*,* PP2C_C_chr02_00030*,* PP2C_C_chr12_01391*,* PP2C_C_chr01_00752*,* PP2C_B_chr13_01243*,* PP2C_A_chr12_00460*,* and PP2C_A_chr01_02277*. In the BR pathway, UW downgraded *BRI1_A_chr11_00260*,* BRI1_B_chr11_01698*,* BAK1_A_chr03_00101*,* BAK1_B_chr06_00603*, and *BSK_C_chr03_01576*, while upgrading *BSK_B_chr06_00056*,* BSK_C_chr06_01576*,* BSK_C_chr06_03682*,* BSK_B_chr06_00122*,* BSK_C_chr06_03763*, and *BSK_C_chr06_03388*. By D4, BL treatment reciprocally modulated gene expression by downregulating UW-upregulated genes (relative to CK) while upregulating UW-downregulated genes compared to CK. By D6, the signs of up-regulation or down-regulation of all the genes between UW and BL were difficult to discern. This could possibly indicate that the sprayed BL had largely lost its protective effect on the camellia oil tree by the time the drought progressed to D6.

### DEGs involved in photosynthesis – antenna proteins

**Fig. 8:**
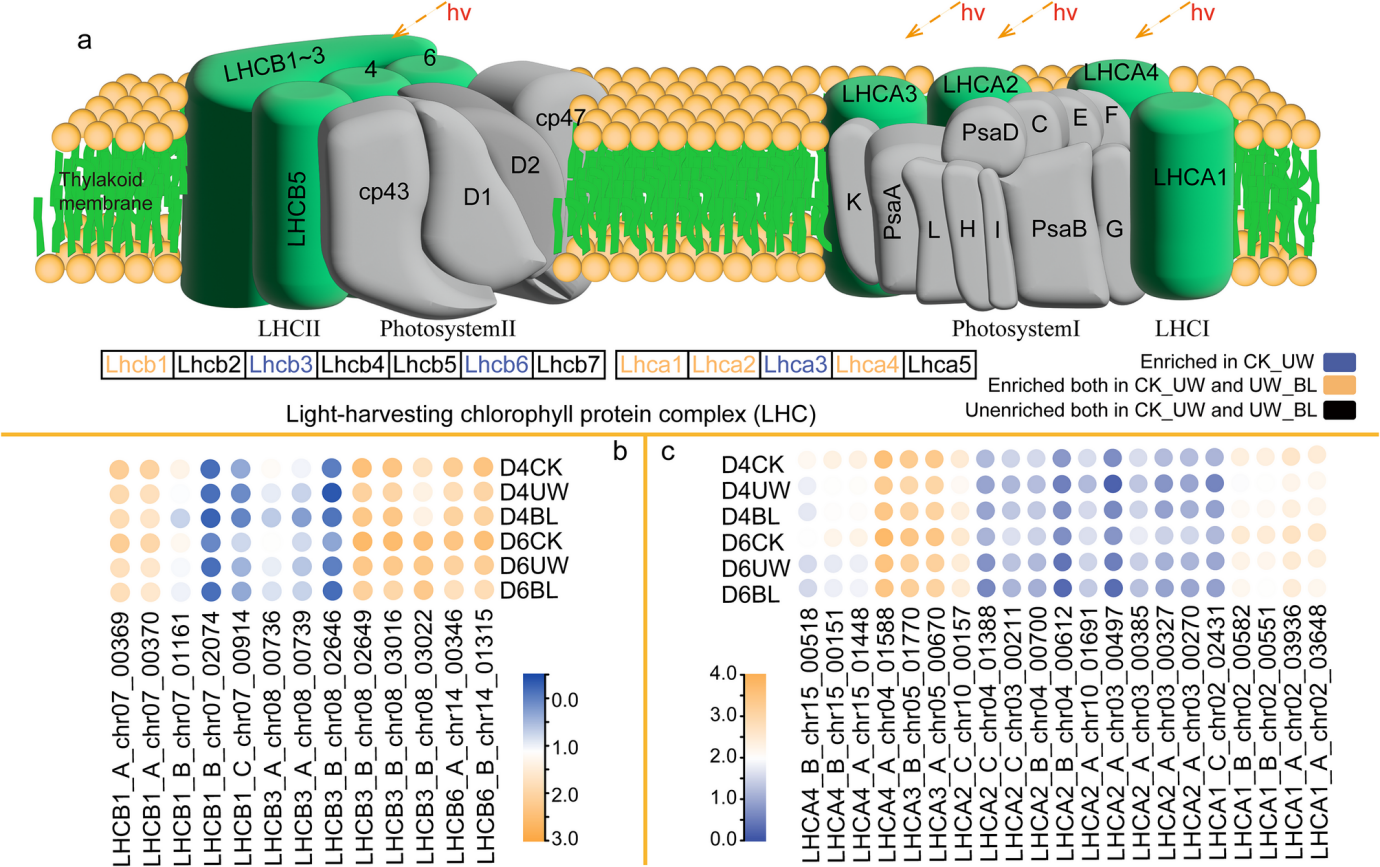
Gene enrichment in photosynthesis-antenna proteins. Note: (**a**) The Light-harvesting chlorophyll protein complex; (**b**) and (**c**) respectively depict the heatmaps of enriched genes in photosystem II and I. The values represented by the colors are FPKM values after normalization (lg)

The photosynthesis-antenna proteins pathway (K00196) was reconstructed to investigate how BL treatment improves photosynthetic efficiency in drought-stressed camellia oil tree leaves (Fig. 8). DEGs in the CK vs. UW comparison were enriched in photosystem I *(Lhca1*,* Lhca2*,* Lhca3*,* Lhca4*) and photosystem II (*Lhcb1*,* Lhcb3*,* Lhcb6*). In contrast, DEGs in the UW vs. BL comparison were primarily enriched in photosystem I (*Lhca1*,* Lhca2*,* Lhca4*) and photosystem II (*Lhcb1*), with *Lhca1*,* Lhca2*,* Lhca4*, and *Lhcb1* shared between both groups. On D4, inverse regulation patterns were observed in photosystem I genes (*LHCA1_A_chr02_03936*,* LHCA1_B_chr02_00551*,* LHCA1_B_chr02_00582*,* LHCA2_B_chr04_00700*,* LHCA2_C_chr03_0021*,* LHCA2_C_chr10_00157*) and photosystem II gene *LHCB3_A_chr08_00736* relative to UW. By D6, BL mirrored UW’s regulation pattern for these genes.

### NAC TFs play a key role in drought mitigation in BL

**Fig. 9:**
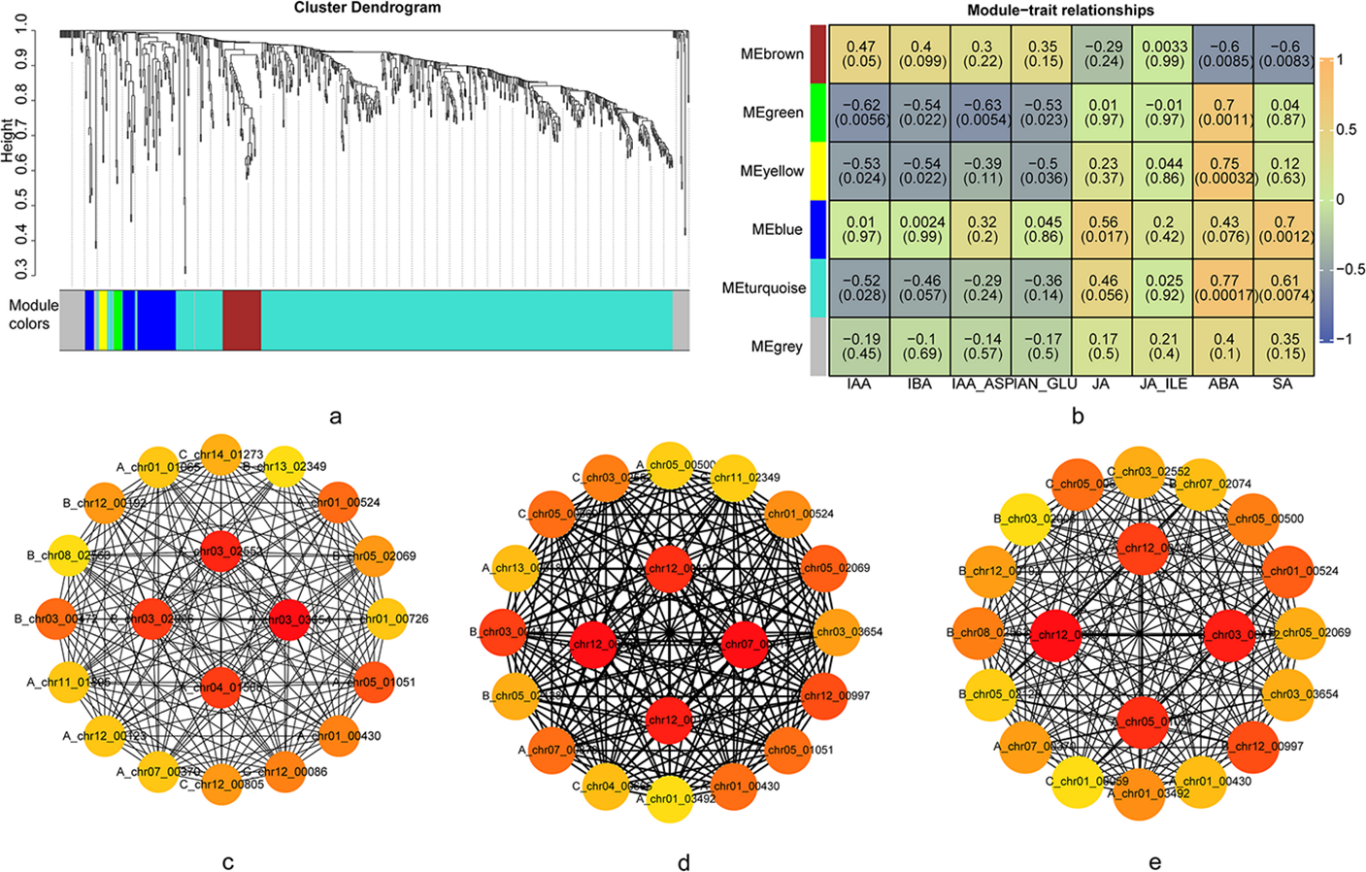
UW vs. BL WGCNA co-expression network. Note: (**a**) Cluster dendrogram of genes subjected to the co-expression module calculation; (**b**) module-trait associations based on Pearson correlations in Camellia oil tree with different hormone; hub genes from green (**c**), yellow (**d**) and turquoise (**e**) modules, the darker the color in c, d, and e, the higher the score

To identify core genes associated with drought resistance following BL application, 4,245 DEGs from the UW vs. BL comparison were analyzed using weighted gene co-expression network analysis (WGCNA). After data preprocessing and removal of under expressed genes, 768 genes were retained for WGCNA. A soft threshold of 28 was selected to maintain a scale-free topology fit (R² ≥ 0.8; Figure S1, S2). The analysis clustered the DEGs into six modules: brown (47 genes), green (10), yellow (10), blue (73), turquoise (569), and grey (59) (Fig. 9a). To explore module-trait relationships, heatmaps were constructed to correlate gene modules with hormone levels. Notably, the green, yellow, and turquoise modules exhibited consistent patterns: negative correlations with auxin-related metabolites (IAA, IBA, IAA_ASP, IAN_GLU) and positive correlations with ABA levels. These three modules were prioritized for further investigation due to their shared regulatory trends (Fig. 9b).

Genes from the green, yellow, and turquoise modules were analyzed in Cytoscape to construct co-expression networks. Using the Maximal Clique Centrality (MCC) algorithm within the cytoHubba plugin, genes were ranked to identify hub genes, with the top 20 prioritized for visualization. In the green module, key hub genes included *C_chr03_02552* (*FRO7*, ferric reduction oxidase 7), *A_chr03_03654* (*FRO7*), *B_chr03_02006* (*PCR2*, plant cadmium resistance 2), and *A_chr04_01588* (*CAB-4*, chlorophyll a-b binding protein P4) (Fig. 9c). The yellow module featured hub genes such as *C_chr07_00914* (hypothetical protein HYC85_029413), *C_chr12_00086* (*uncharacterized protein LOC114260969*), *B_chr12_00192* (*hypothetical protein LOK49_LG13G00242*), and *A_chr12_00123* (*uncharacterized protein LOC114314370*) (Fig. 9d). In the turquoise module, prominent hub genes comprised *C_chr12_00086* (u*ncharacterized protein LOC114260969*), *B_chr03_00472* (*FRO7*), *A_chr05_01051* (*OMT3*, O-methyltransferase 3), and *A_chr12_00123* (*uncharacterized protein LOC114314370*) (Fig. 9e).

**Fig. 10:**
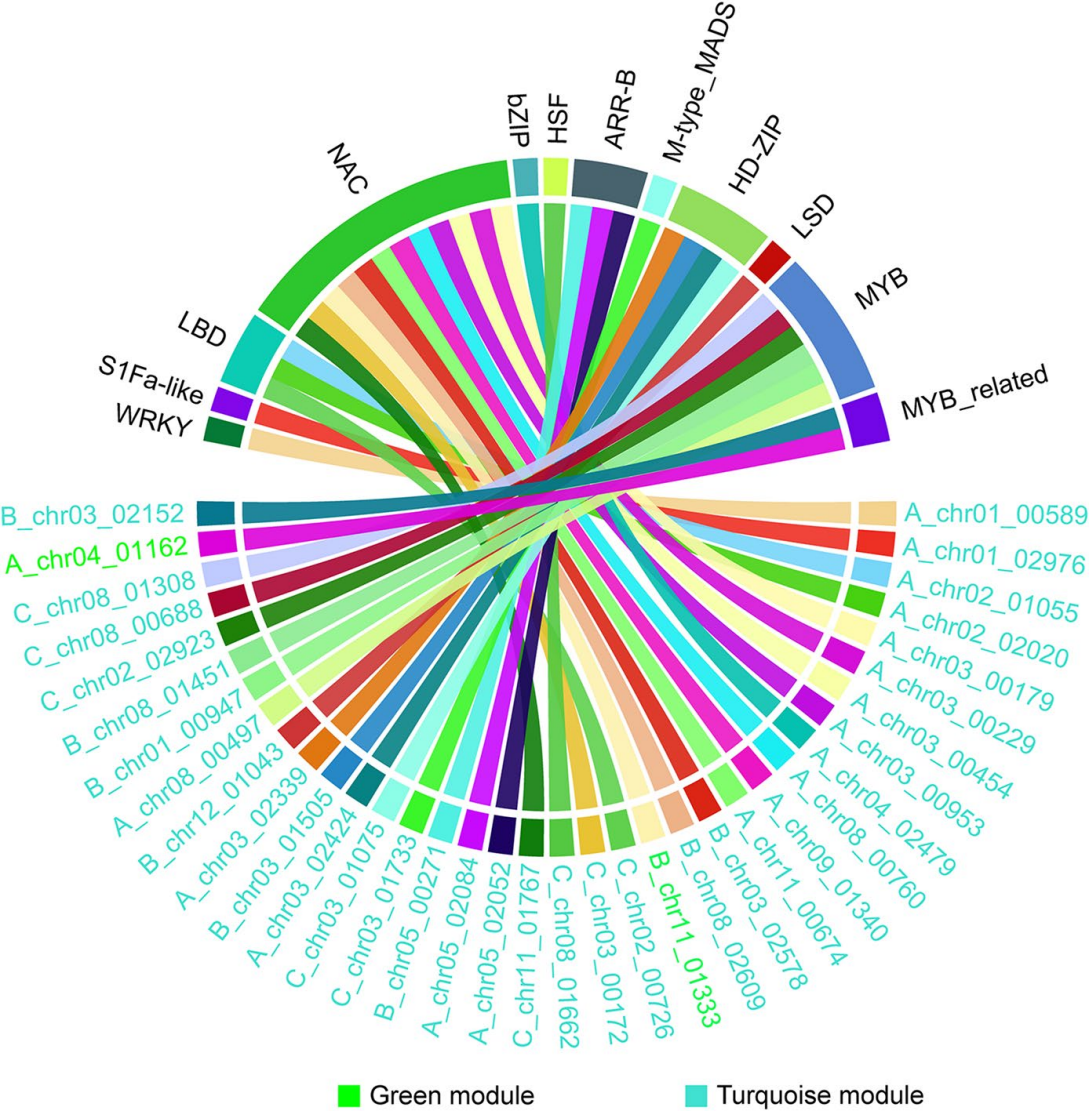
Transcription Factors Corresponding to blue, green and turquoise modules. Note: The different colors of the fonts represent the modules to which they belong, and no TFs belong to yellow module

Subsequently, sequences from these modules were submitted to PlantTFDB (https://planttfdb.gao-lab.org/prediction.php) for transcription factor (TF) prediction. Among 589 genes analyzed, 36 TFs were identified (Fig. 10). The most frequent families included NAC (12 occurrences), MYB (6), HD-ZIP (4), LBD (3), ARR-B (3), and MYB-related (2), with WRKY, S1Fa-like, bZIP, HSF, M-type MADS, and LSD each appearing once. The predominance of NAC transcription factors suggests their critical regulatory role in mitigating drought stress in Camellia oleifera.

### Validation of RNA-seq results through q-PCR analysis of drought stress response-related genes in Camellia oil tree

**Fig. 11:**
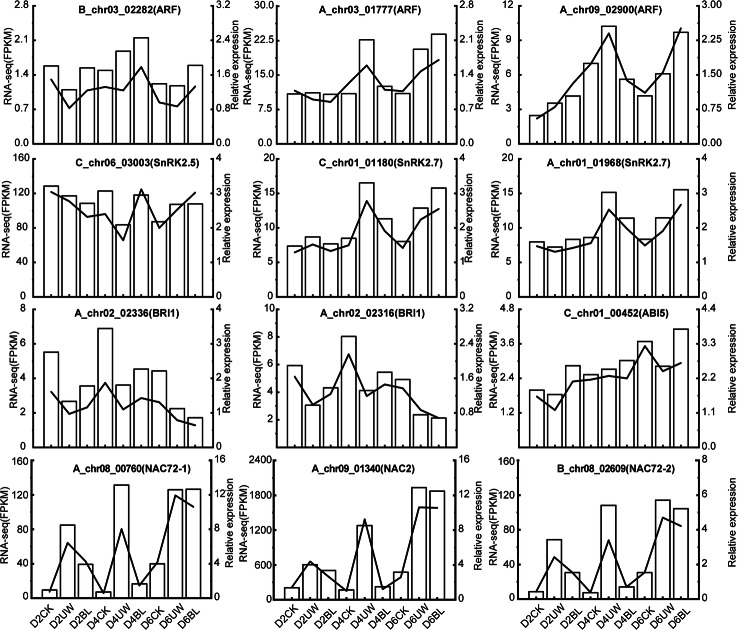
Quantitative q-PCR verification of nine candidate genes related to drought. Note: The left axis is RNA-seq (FPKM) corresponding to the bar graph in the figure; the right axis is the relative expression calculated from the results of qRT-PCR, corresponding to the line graph in the figure

To validate the reliability of the RNA-seq and further elucidate the pathway by which BL enhances drought resistance in Camellia oil tree, twelve drought stress response-related genes were analyzed via q-PCR. These included three SnRK2 genes (*C_chr06_03003*,* C_chr01_01180*,* A_chr01_01968*), three auxin response factor (ARF) genes (*B_chr03_02282*,* A_chr03_01777*,* A_chr09_02900*), one abscisic acid-insensitive 5 (ABI5) gene (*C_chr01_00452*), two brassinosteroid-insensitive 1 (BRI1) genes (*A_chr02_02316*,* C_chr01_00452*), and three NAC-related genes (*A_chr08_00760*,* A_chr09_01340*,* B_chr08_02609*). The qRT-PCR results exhibited expression patterns consistent with the RNA-seq data, confirming the high reliability of the transcriptome dataset (Fig. 11).

## Discussion

### Response of leaves to drought

The leaf serves as the principal organ for photosynthesis, with its functional efficiency becoming most apparent under drought stress. Studies demonstrate that drought significantly inhibits leaf photosynthetic rates. Re-examination of the photosynthetic apparatus revealed downregulation of LHC genes under drought conditions. Conversely, BL application upregulated multiple LHC genes. In higher plants, the photosynthetic machinery is primarily composed of PSI and PSII. The PSI antenna complexes are encoded by six genes (*Lhca1–Lhca6*) [41], with *Lhca1–Lhca4* being critical for assembling the antenna system and integrating into the PSI core complex. The PSII antenna complexes are encoded by *Lhcb1–Lhcb7* genes. Both photosystems function to capture light energy efficiently and transfer it through subsequent photosynthetic stages [42]. Notably, BL-treated plants maintained robust light-harvesting capacity, which likely preserved photosynthetic rates under drought stress.

Meanwhile, drought exerted limited effects on BL-treated leaves. Anatomical analysis revealed that UW leaves exhibited significantly reduced leaf thickness, spongy tissue thickness, and palisade tissue thickness compared to CK plants across all time points, while the palisade-to-spongy tissue ratio progressively increased. BL-treated plants displayed analogous anatomical changes from D4 onward. These morphological responses may be attributed to drought-induced stomatal regulation. Under water scarcity, plants initiate stomatal closure to minimize water loss. This adaptive mechanism, however, restricts carbon dioxide influx into leaves, directly limiting photosynthetic substrate availability and consequently reducing photosynthetic efficiency [43]. Prolonged drought exacerbates water deficits, as insufficient soil moisture fails to replenish transpirational losses, ultimately inducing leaf dehydration and structural collapse [9]. Notably, the spongy tissue—characterized by its loosely arranged cells—is disproportionately vulnerable to drought stress. Chronic water deprivation further disrupts the compact architecture of palisade tissue, culminating in overall leaf thickness reduction.

Drought stress induces an increase in ABA levels in plants as an adaptive response to environmental challenges, a finding consistent with previous studies [44]. Notably, in leaves treated with exogenous BL, the elevation of ABA content was not observed on D2 but became evident on D4. There might be two theories to explain this phenomenon. First, this suggests that BL application may trigger other signaling molecules that mediate drought stress responses earlier than ABA. Experimental data from this study indicate that JA is likely the first phytohormone to respond during the initial phase of drought stress [45–47]. During early drought exposure, Camellia oil tree may activate its defense system by increasing JA levels, prioritizing enhanced stress resistance as the primary survival strategy [48]. As drought persists, the plant shifts to synthesizing substantial amounts of ABA to reinforce defense mechanisms, dynamically balancing growth and stress adaptation through metabolic regulation [48–50]. Second, although the ABA content in leaves began to increase on D4 after BL application, this does not necessarily indicate that BL promotes endogenous ABA accumulation [51–54]. The enhancement of drought resistance may be achieved through ABA-independent pathways [6, 55]. Previous studies have shown that exogenous BL can increase BRs content in plants [56], but this study could not conclusively determine how BL influences drought response at the molecular level.

Previous study found that the expression of *IAA5*,* IAA6*, and *IAA19*—negative regulators of growth hormones—is required in plants under stress conditions [57]. This suggests that plants may reduce internal IAA levels to maintain basal survival under stress. Interestingly, in this study, the BL treatment resulted in even lower IAA-like hormone content than the UW treatment, although the difference was not statistically significant. This phenomenon may be related to the fact that BR and auxin share a substantial number of precursor synthesizing enzymes [58]. When BL was applied to induce BR synthesis in plants, a considerable portion of precursor substances may have been diverted to BR biosynthesis, thereby reducing IAA synthesis [59].

### NAC transcription factor response to drought stress

**Fig. 12:**
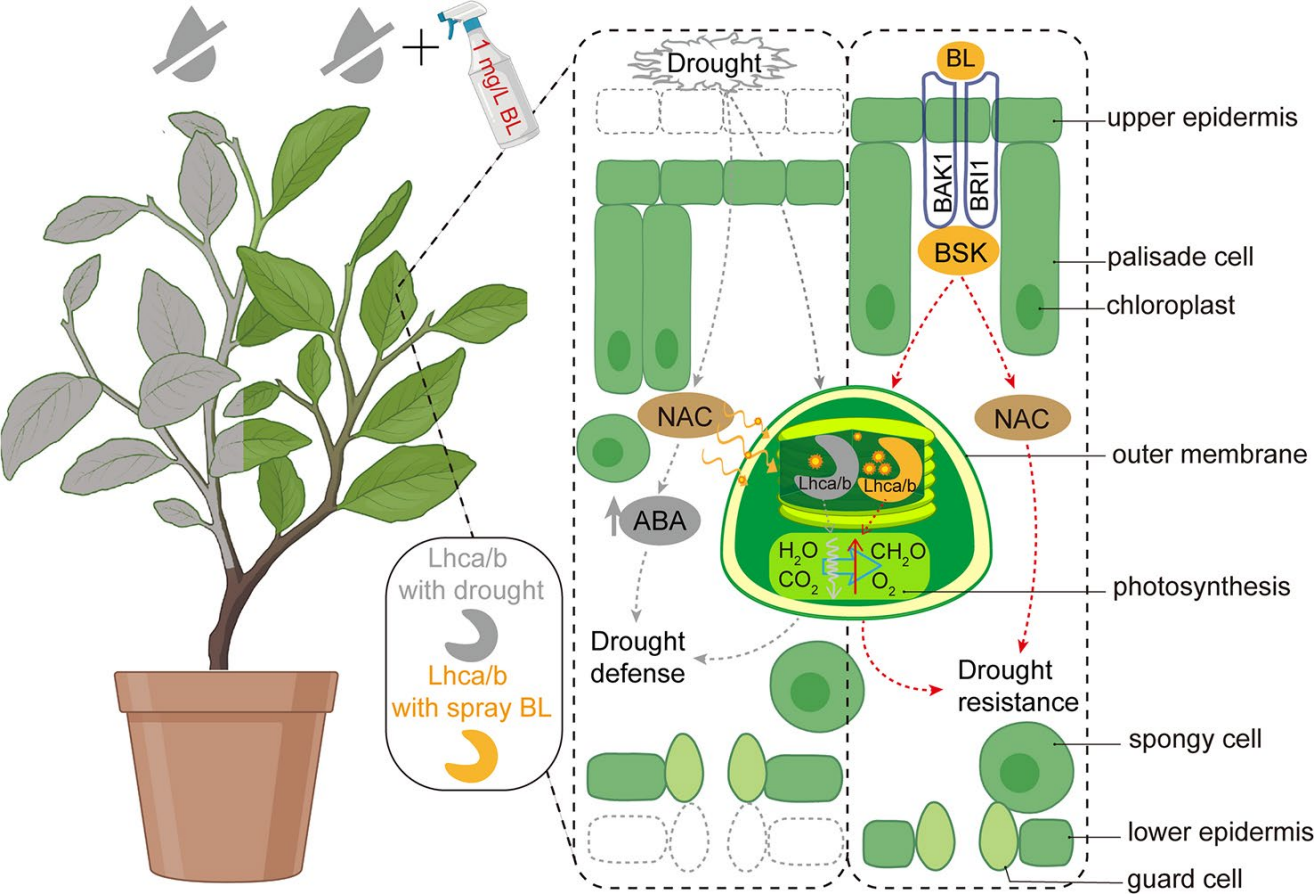
Schematic diagram of BL mitigation drought for Camellia oil tree. Note: Drought stress induces leaf shrinkage and thinning (the dashed box indicates the normal epidermal region of the leaf). The red dotted lines illustrate potential pathway through which BL application alleviates drought stress. Compared to drought treatment alone, BL-sprayed leaves exhibit enhanced light capture capacity and reduced inhibition of photosynthetic rate under drought conditions. Concurrently, NAC TFs may play a critical role in BL-mediated drought mitigation

NAC TFs play pivotal roles in plant drought stress responses [60, 61]. In this study, we observed significant downregulation of NAC family genes in Camellia oleifera under BL treatment compared to UW conditions, suggesting their potential role as key regulators in drought stress mitigation. However, due to the functional diversity within the NAC family, their drought regulatory mechanisms are not exclusively ABA-dependent [62]. For instance, in Arabidopsis, AtNAC72 (RD29), AtNAC109, and AtNAC55 enhance drought tolerance by promoting aldehyde detoxification via the glyoxalase pathway [63]. In rice, overexpression of *SNAC1*, *OsNAC5*, *OsNAC6*, and *OsNAC10* improves both drought tolerance and yield [64]. Similarly, *VuNAC1/2* optimizes the balance between carbon assimilation and water-use efficiency by modulating stomatal activity in cowpea [65]. Parallel mechanisms have been reported in maize and grapevine, where NAC family members regulate stomatal aperture, closure, and density under drought stress [66, 67]. A key limitation of this study lies in the absence of comprehensive physiological profiling to definitively characterize BL-mediated drought alleviation pathways; thus, interpretations rely on partial experimental data and comparative analyses with existing findings. Based on these observations, we propose a schematic model (Fig. 12) illustrating two hypothesized mechanisms: (1) BL enhances photosynthetic efficiency by improving light capture in drought-stressed Camellia oil tree leaves, and (2) NAC TFs may contribute to BL-induced drought tolerance through ABA-independent pathways.

While this study addressed our initial hypotheses—confirming that BL alleviates drought stress and implicating the NAC family as a key player in this process—it has raised additional unresolved questions: Is the observed increase in ABA content directly linked to BL application? If not, through what mechanisms does BL mitigate drought stress? What precise role does NAC TFs play in mediating interactions among BL, ABA, and drought resistance? These questions require deeper molecular biological investigations to elucidate the underlying pathways and regulatory networks, which will constitute the primary focus of our research groups subsequent investigations.

## Conclusion

This study investigated the effects of BL on leaf anatomical structure and hormone content under drought stress in Camellia oil tree. Results demonstrated that BL application effectively mitigates drought stress, primarily through enhanced light capture efficiency and reduced ABA accumulation. WGCNA revealed that a significant number of differentially expressed genes are associated with NAC transcription factors, suggesting a potential role of NACs in BL-induced regulation of ABA-related responses.

## Electronic supplementary material

Below is the link to the electronic supplementary material.

Supplementary Material 1

## Data Availability

The raw sequence data reported in this paper have been deposited in the Genome Sequence Archive (Genomics, Proteomics & Bioinformatics 2021) in National Genomics Data Center (Nucleic Acids Res 2022), China National Center for Bioinformation / Beijing Institute of Genomics, Chinese Academy of Sciences (GSA: CRA021215) that are publicly accessible at https://ngdc.cncb.ac.cn/gsa.
