# Supplementary material for: NAC transcription factors are key regulators of Brassinolide-Enhanced drought tolerance in Camellia oil tree
Source: BMC Plant Biol. 2025 May 13;25:625. doi: 10.1186/s12870-025-06653-0 (PMC12070694; doi:10.1186/s12870-025-06653-0)
Supplement: Supplementary file 1 — Supplementary Material 1 [file 12870_2025_6653_MOESM1_ESM.docx]

Table S1 Temperature during the experiment

| **Date** | **Average Temperature ℃** | **Highest**  **Temperature ℃** | **Minimum**  **Temperature ℃** |
| --- | --- | --- | --- |
| Day1 | 32.1 | 39.1 | 27.4 |
| Day2 | 32.8 | 38.2 | 26.7 |
| Day3 | 33.3 | 39.7 | 26.5 |
| Day4 | 28.6 | 39.7 | 25.4 |
| Day5 | 30.3 | 37.3 | 25.3 |
| Day6 | 31.9 | 38.0 | 25.3 |

Table S2 Soil water content during the experiment

| **Treatments** | **Soil water content %** | | |
| --- | --- | --- | --- |
|  | **D2** | **D4** | **D6** |
| CK | 27.14 | 26.22 | 28.19 |
| UW | 16.27 | 11.90 | 9.59 |
| BL | 17.65 | 12.64 | 9.13 |

Table S3 Sequence information of gene-specific primers of 9 genes for qRT-PCR analyses

|  | **Gene ID** | **Description** | **Forward Primer (5' to 3')** | **Reverse Primer (5' to 3')** |
| --- | --- | --- | --- | --- |
| 1 | B_chr03_02282 | auxin response factor (ARF) | CGCACAACACCAGAACAAGG | TGCATTACGGGCTTTCGAGT |
| 2 | A_chr03_01777 | auxin response factor (ARF) | CGGGTTGCCGATGCTTATTG | AGACAGACAACAGAGCCTGC |
| 3 | A_chr09_02900 | auxin response factor (ARF) | CCGAGACCGTGTAGTTGAGG | TACAGCCCTTCTCCGGATGT |
| 4 | C_chr06_03003 | sucrose nonfermenting 1(SNF1)-related protein kinase 2.5 (SnRK2.5) | TGAGCTCGTTGCTGTCAAGT | CTCACTGAACCGTCCTGCAT |
| 5 | C_chr01_01180 | sucrose nonfermenting 1(SNF1)-related protein kinase 2.7 (SnRK2.5) | GCTGACCCTGCAAAGAGGAT | CATGGGTTGATCGGGCTCTT |
| 6 | A_chr01_01968 | sucrose nonfermenting 1(SNF1)-related protein kinase 2.7 (SnRK2.5) | GCTGACCCTGCAAAGAGGAT | CATGGGTTGATCGGGCTCTT |
| 7 | A_chr02_02336 | brassinosteroid-insensitive1 (BRI1) | ATGGGACTATCCCACCTGCT | CGGCCTGATTCCTCCAAACT |
| 8 | A_chr02_02316 | brassinosteroid-insensitive1 (BRI1) | TTTCTACGGTGCGGTTCCTC | GCGATGAAATTGGCAGCGAT |
| 9 | C_chr01_00452 | basic-leucine zipper (bZIP) transcription factor family protein (ABI5) | CGACAGATAGGGGCATTGCT | CGTCCACCGTTTTTCGACAC |
| 10 | A_chr08_00760 | NAC72-1 | GGTTCAGAGCGGACACAGAA | ACGGGTCAGGCGAATTACTG |
| 11 | A_chr09_01340 | NAC2 | GCTCCGAGGGGAGAGAAAAC | TGCGACATAGGACCCAATCG |
| 12 | B_chr08_02609 | NAC72-2 | GGTTCAGAGCGGACACAGAA | ACGGGTCAGGCGAATTACTG |

**
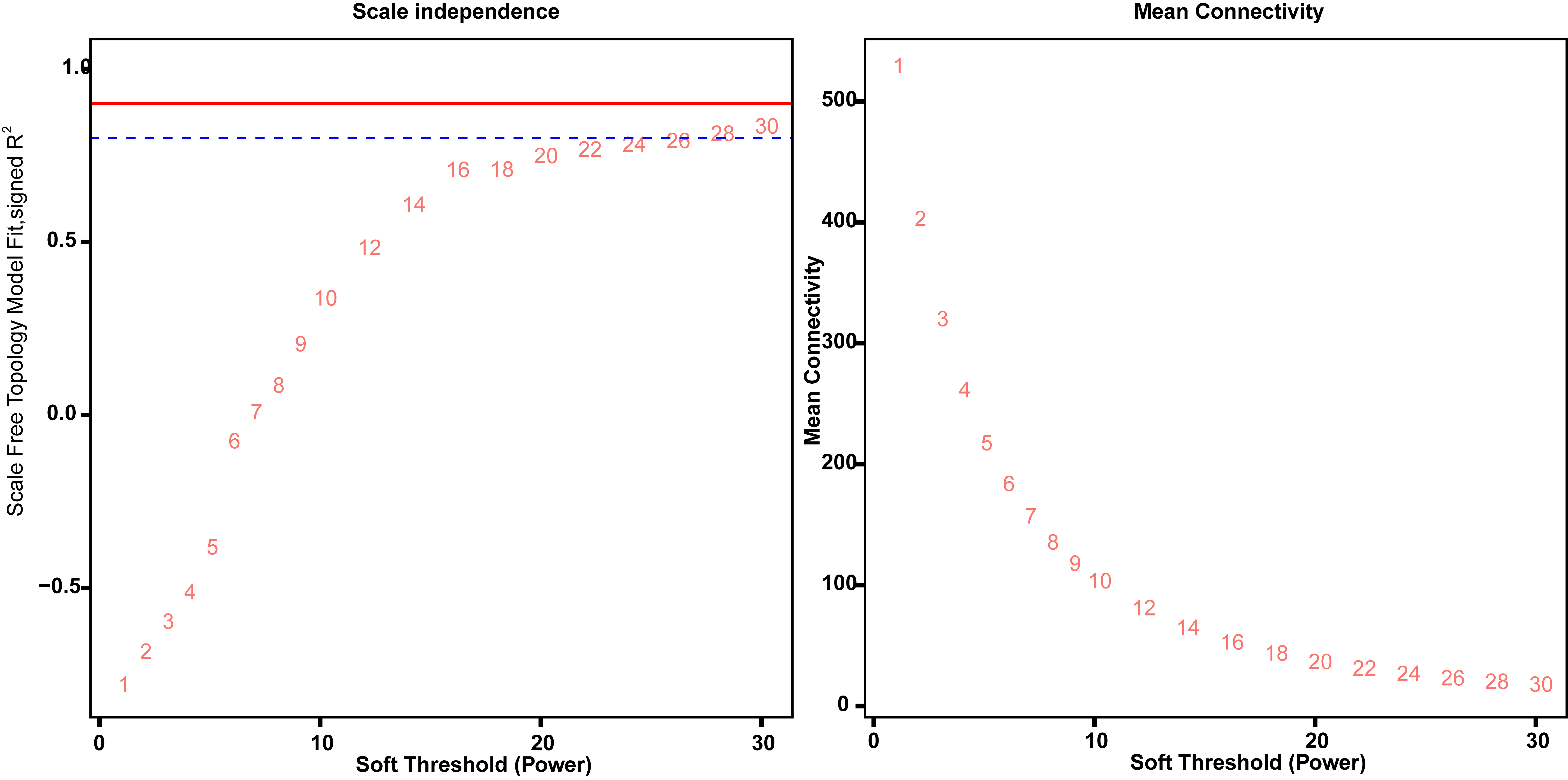
**

Figure S1 Soft threshold set up

**
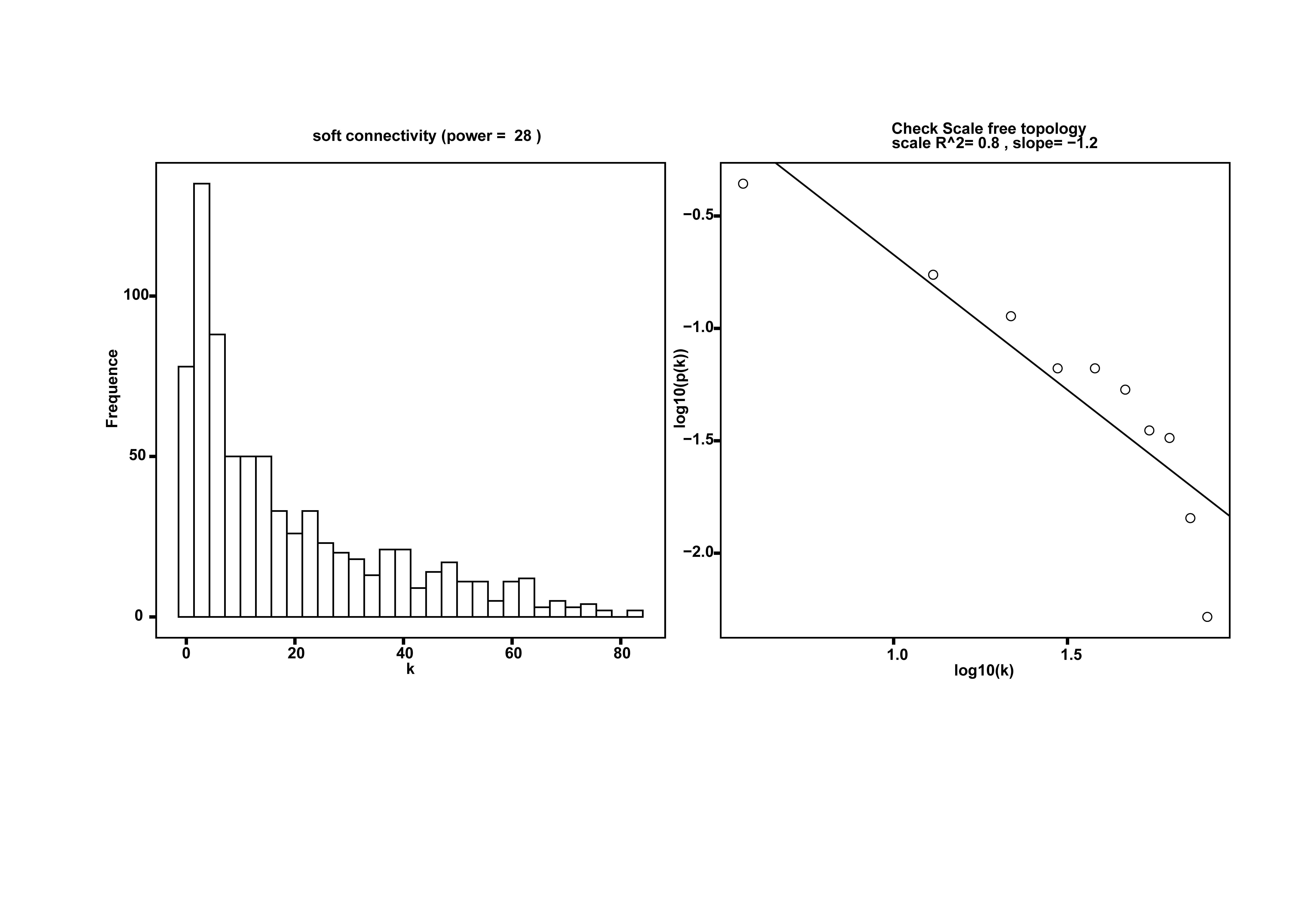
**

Figure S2 Soft threshold check
